# The Effects of Frondanol, a Non-Polar Extract of the Atlantic Sea Cucumber, in Colon Cancer Cells

**DOI:** 10.3390/ph18111714

**Published:** 2025-11-11

**Authors:** Hardik Ghelani, Hala Altaher, Hadil Sarsour, Marah Tabbal, Sally Badawi, Thomas E. Adrian, Reem K. Jan

**Affiliations:** College of Medicine, Mohammed Bin Rashid University of Medicine and Health Sciences, Dubai Health, Dubai P.O. Box 505055, United Arab Emirates; hardik.ghelani@dubaihealth.ae (H.G.); hala.taher@students.mbru.ac.ae (H.A.); hadil.sarsour@students.mbru.ac.ae (H.S.); marah.altabbal@alumni.mbru.ac.ae (M.T.); sally.badawi@dubaihealth.ae (S.B.); thomas.adrian@dubaihealth.ae (T.E.A.)

**Keywords:** 5-lipoxygenase, apoptosis, *Cucumaria frondosa*, cytotoxicity, colorectal cancer, Caco-2, Frondanol, HT-29

## Abstract

**Background:** Colorectal cancer (CRC) is the second leading cause of cancer-related mortality worldwide. The search for effective, new antineoplastic drugs with fewer side effects for the treatment of CRC continues, with marine-derived compounds emerging as promising candidates. **Objectives:** This study investigates the anticancer potential of Frondanol, a nutraceutical derived from the Atlantic Sea cucumber *Cucumaria frondosa*, known for its potent anti-inflammatory properties. **Methods:** Two human CRC cell lines, Caco-2 and HT-29, were used to test the effects of Frondanol using various in vitro approaches. **Results:** Frondanol significantly inhibited cell viability in a dose- and time-dependent manner. At a 1:10,000 dilution, viability decreased to around 30% in Caco-2 and 20% in HT-29 after 24 h, dropping to nearly 5% at 48 h. Furthermore, a clonogenic assay showed around 50% reduction in colony formation in both cell lines. Flow cytometry-based Annexin V staining revealed that Frondanol increased early apoptosis to ~5.2% in Caco-2 and ~9.4% in HT-29 cells, while cell cycle analysis showed accumulation of the sub G0 (apoptotic) phase increasing from 1.5% to 14.7% (Caco-2) and from 1.9% to 23.8% (HT-29). At the molecular level, Frondanol treatment significantly decreased anti-apoptotic protein B-cell lymphoma (Bcl)-2 expression while increasing the expression of the proapoptotic protein Bcl-2-associated X-protein. Additionally, Frondanol markedly induced cytochrome c release from the mitochondria and activated caspase-9, caspase-7, and caspase-3 after treatment, alongside cleavage of the caspase-3 substrate poly (ADP-ribose) polymerase. Frondanol inhibited 5-lipoxygenase activity, further contributing to its anticancer effects. **Conclusions:** In conclusion, Frondanol inhibits CRC cell proliferation and induces apoptosis through the mitochondrial pathway in vitro, suggesting that it is a potential nutraceutical for the prevention of human colorectal cancer or a valuable source of anticancer compounds.

## 1. Introduction

Colorectal cancer (CRC) usually arises from adenomatous polyps or flat adenomas in the colon or rectum, progressing through the accumulation of genetic mutations and dysregulated cell growth that ultimately leads to uncontrolled proliferation and tumor formation [1]. According to recent epidemiological data, it is estimated that there were approximately 1.9 million new cases and 930,000 deaths related to CRC worldwide in 2020. The burden of CRC is expected to increase, with projections indicating that by 2040 the number of new cases will rise to about 3.2 million new cases and 1.6 million deaths annually [2]. The rising incidence rates and the complex interplay of genetic, environmental, and lifestyle factors underscore the urgency for effective therapeutic strategies against this disease [3].

Both genetic and environmental factors influence the initiation and progression of CRC. While less than 10% of CRC cases are due to inherited syndromes such as familial adenomatous polyposis and Lynch syndrome, the majority are sporadic. Lifestyle factors such as diet, physical activity, smoking, and alcohol consumption also play significant roles [4,5]. Moreover, chronic inflammatory conditions in the colon, including inflammatory bowel disease (ulcerative colitis and Crohn’s disease), contribute significantly to the augmented risk of developing CRC [6]. Current treatments for CRC primarily involve a combination of surgery and chemotherapy, although significant challenges often accompany them. While surgery targets tumor removal and affected tissues, it may not always be curative, especially in advanced stages or cases of metastasis [7]. Moreover, despite their therapeutic efficacy, chemotherapeutic drugs, such as fluorouracil, oxaliplatin, and irinotecan, despite their therapeutic efficacy, are associated with adverse effects and the development of drug resistance over time [8]. Although advancements in screening, diagnosis, and treatment have been made, CRC remains a significant cause of morbidity and mortality worldwide. Prevention efforts focusing on lifestyle modifications, early detection through screening programs, and targeted therapeutic interventions are crucial in mitigating the burden of CRC and improving patient outcomes [9]. In light of these challenges, exploring novel preventive and therapeutic agents such as natural compounds warrants investigation, offering potential insights into alternative approaches for combating CRC.

Frondanol, a nutraceutical extract of the edible sea cucumber *Cucumaria frondosa*, is a US-patented and reported to possess potent anti-inflammatory activity, confirmed in animal and human studies without signs of toxicity [10,11,12]. In a mouse model of colitis, treatment with Frondanol significantly reduced colonic inflammation and levels of inflammatory cytokines such as tumor necrosis factor (TNF)-α, interleukin (IL)-6, and IL-1β, indicating its potential anti-inflammatory action in the gut lining [11]. Based on these promising preclinical results, a randomized, double-blind, placebo-controlled clinical trial was initiated to assess Frondanol’s effectiveness in adults with inflammatory bowel disease (IBD) [10]. The trial is nearing completion, and preliminary data suggest that Frondanol exerts anti-inflammatory effects in IBD patients, aligning with the outcomes observed in animal studies. Additionally, in vitro experiments have demonstrated that Frondanol inhibits the 5-lipoxygenase (5-LOX) and 12-lipoxygenase (12-LOX) pathways, leading to reduced production of inflammatory lipid mediators such as 12-HETE, 5-HETE, and leukotriene B4 (LTB_4_) in human neutrophils [12]. Moreover, recent findings from our laboratory, demonstrate that Frondanol attenuates inflammation in LPS-induced RAW 264.7 macrophages through modulation of NF-κB and MAPK signaling pathways [13].

Despite the substantial evidence supporting Frondanol’s anti-inflammatory activity, its potential as an anticancer agent in CRC remains unexplored. Given the intricate interplay between inflammation and cancer progression, there exists a compelling hypothesis that Frondanol’s anti-inflammatory effects may extend to inhibition of CRC cell growth and proliferation [14,15]. This study primarily aims to investigate Frondanol’s anticancer potential in CRC and to further explore the mechanisms through which it induces apoptosis in human colorectal cancer cells and impacts CRC growth using an in vitro approach.

Preliminary findings suggest that Frondanol triggers cytochrome c release and activates the caspase cascade, implicating the involvement of the anti-apoptotic, pro-apoptotic, and, subsequently, caspase proteins in this process. Using several in vitro analyses, this study seeks to provide valuable insights into the anticancer potential of Frondanol, offering new avenues for developing natural therapeutic strategies against CRC.

## 2. Results

### 2.1. Fatty Acid Profiling of Frondanol

Fatty acid profiling of Frondanol revealed the highest concentrations of fatty acids to be 12-methyltetradecanoic acid (12-MTA, 19.6%), followed by eicosapentaenoic (EPA, 17.9%), palmitoleic acid (12.7%), oleic acid (4.49%), stearic acid (2.84%), myristoleic acid (2.71%), myristic (2.54%), linoleic acid (2.54%) and docosahexaenoic acid (DHA, 0.6%) (Table 1). The overall composition included 9.52% saturated fatty acids, 21.68% monounsaturated, and 23.73% polyunsaturated fatty acids (Table 2). This bioactive lipid profile revealed that it is rich in the branched–chain fatty acid (12-MTA) as well as long-chain polyunsaturated fatty acids such as EPA and DHA, all of which are known for their immunomodulatory and anti-inflammatory properties.

### 2.2. Frondanol Suppressed Colon Cancer Cell Proliferation in a Dose-Dependent Manner

**Figure 1:**
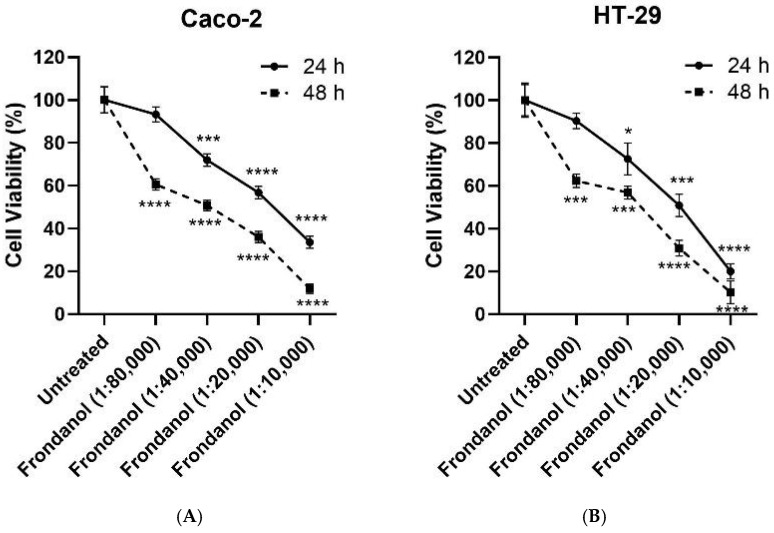
Effects of Frondanol on the viability of Caco-2 and HT-29 cells. The percentage cell viability of (**A**) Caco-2 and (**B**) HT-29 colorectal cancer cells following Frondanol treatment. Each point in the line graph represents the mean ± SEM of three replicate experiments. * *p* < 0.05, *** *p* < 0.001 and **** *p* < 0.0001 in comparison to untreated cells.

**Figure 2:**
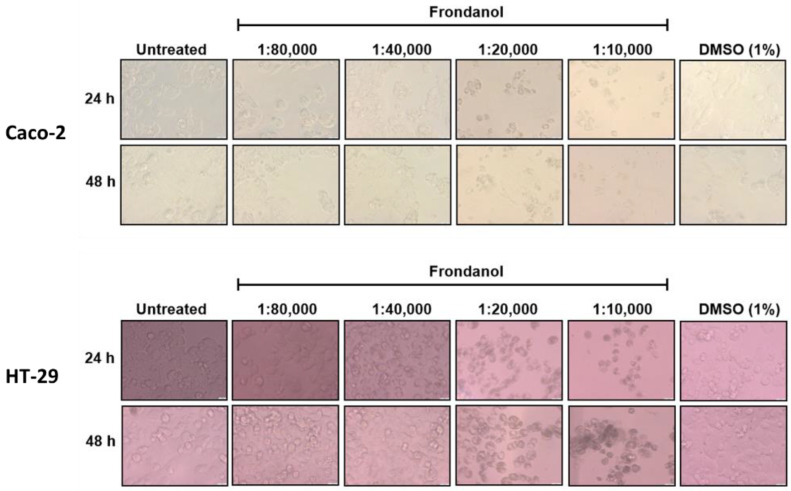
Effect of Frondanol on the morphology of Caco-2 and HT-29 cells. The observed changes in colorectal cancer cells include morphological alterations supportive of apoptosis, such as shrinkage of the cell body, rounding, and detachment from the substrate, as observed under 40× magnification.

The antiproliferative effects of Frondanol on HT-29 and Caco-2 cells were assessed using the CellTiter-Glo Luminescent Cell Viability assay, which measures ATP levels as an indicator of cell viability. Our results suggested a dose-dependent cell growth inhibitory effect of Frondanol on both CRC cells (Figure 1 and Figure 2). Frondanol demonstrated significant antiproliferative effects against both CRC cells, with IC_50_ values of approximately 1:20,000 (0.005% *v*/*v*) and 1:40,000 (0.0025% *v*/*v*) at 24 and 48 h, respectively (Figure 1). Following 24 and 48 h of Frondanol treatment, morphological alterations were photographed through light microscopy. Compared to untreated cells and those treated with DMSO (1%) vehicle control, Frondanol-treated cells exhibited notable changes in morphology, including reduced cell confluence and a shift towards non-adherent, rounded shapes. These morphological alterations were particularly pronounced at higher concentrations of Frondanol, with cells treated at the highest concentration displaying detached and rounded morphology (Figure 2).

### 2.3. Frondanol Reduced CRC Cell Lines’ Clonogenic Ability

**Figure 3:**
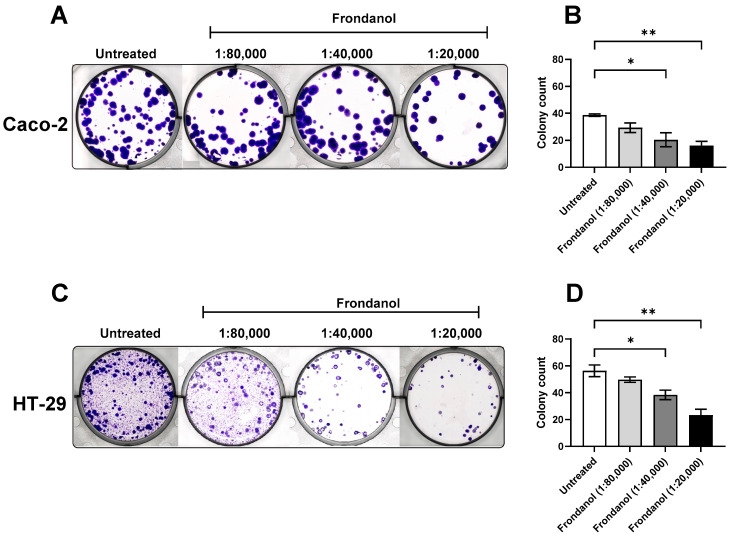
Effects of Frondanol on the colony formation of Caco-2 and HT-29 cells. (**A**,**C**) Representative photograph of colony formation in six-well plates for Caco-2 and HT-29 cells, (**B**,**D**) Number of colonies formed by Caco-2 and HT-29 cells in the absence (untreated) and presence of increasing concentrations of Frondanol. Each bar represents the mean ± SEM of three replicate experiments. * *p* < 0.05, ** *p* < 0.01 when compared to untreated.

To evaluate the anticancer properties of Frondanol on CRC cells, a colony formation assay (clonogenic assay) was performed. This assay evaluates the ability of individual cells to form colonies, providing insights into their proliferative capacity. The quantitative analysis of colony counts revealed a significant and dose-dependent effect of Frondanol on colony growth (Figure 3). Treatment with Frondanol at a dilution of 1:40,000 resulted in a marked reduction in colony formation, with a decrease of more than 30% compared to untreated cells. Moreover, treatment with a higher concentration (1:20,000 dilution) of Frondanol demonstrated enhanced efficacy, resulting in a significant reduction of approximately 60% in colony formation compared to untreated Caco-2 and HT-29 cells.

### 2.4. Frondanol Induced Apoptosis in AO/EtBr Staining Assay

**Figure 4:**
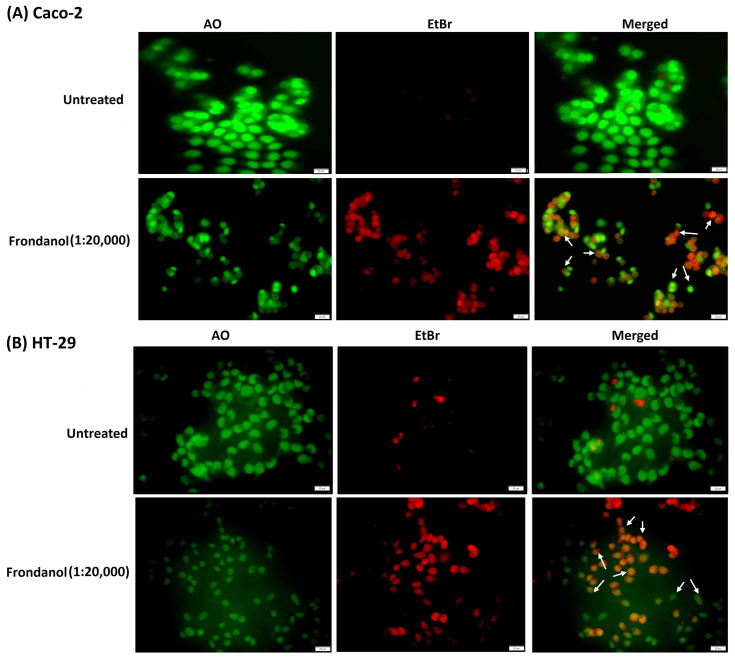
Acridine orange/ethidium bromide staining of colorectal cancer cells (**A**) Caco-2 and (**B**) HT-29. The cells treated with Frondanol (1:20,000 dilution) showed red and orange fluorescence, indicating cells undergoing apoptosis (white arrows).

The dual acridine orange/ethidium bromide (AO/EtBr) double-staining technique has been widely used to detect cell morphological changes during apoptosis. Acridine orange is a vital dye that stains live and dead cells and gives a green fluorescence. In contrast, ethidium bromide only stains cells that have lost their membrane integrity and provide a red fluorescence. Cells were visualized under the microscope as viable cells (green nuclei), apoptotic cells (nuclei condensed and orange color), and dead cells (red nuclei). Our results indicated that Frondanol (at a dilution of 1:20,000) diminished cell viability and promoted apoptosis in Caco-2 and HT-29 cancer cells. As depicted in Figure 4, the living cells appear green, while apoptotic cells with orange particles in the nucleus are distinguished.

### 2.5. Frondanol Induced Apoptosis in Hoechst 33342 Staining Assay

**Figure 5:**
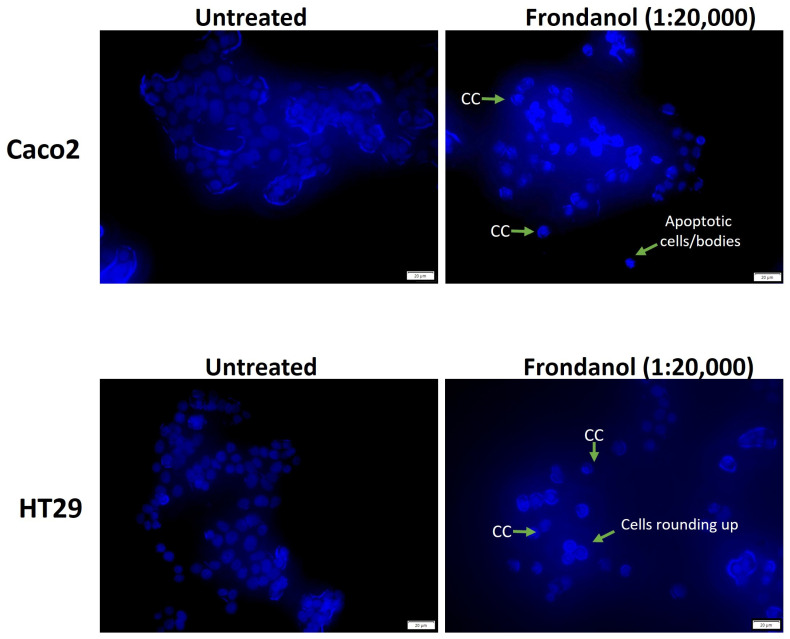
The morphological changes of cancer cells (Caco-2 and HT-29) were detected with Hoechst 33342 staining. Cells were treated with Frondanol (1:20,000) for 48 h and imaged by fluorescence microscope (magnification 40×). Arrows indicate chromatin condensation (CC), rounded cells, and apoptotic cells/bodies.

Morphological examination of cell nuclei in Caco-2 and HT-29 cells after 48 h of treatment, with or without Frondanol (at a dilution of 1:20,000), revealed significant changes compared to untreated cells. As depicted in Figure 5, untreated cells displayed an intact oval shape with nuclei exhibiting a relatively faint blue fluorescence, indicative of staining with Hoechst 33342 dye. In contrast, cells treated with Frondanol exhibited characteristic signs of apoptosis, including cell rounding, shrinkage, chromatin condensation (CC), the formation of apoptotic bodies, and reduced cell numbers.

### 2.6. Frondanol Induced Early Apoptosis in Annexin V-PI Assay

**Figure 6:**
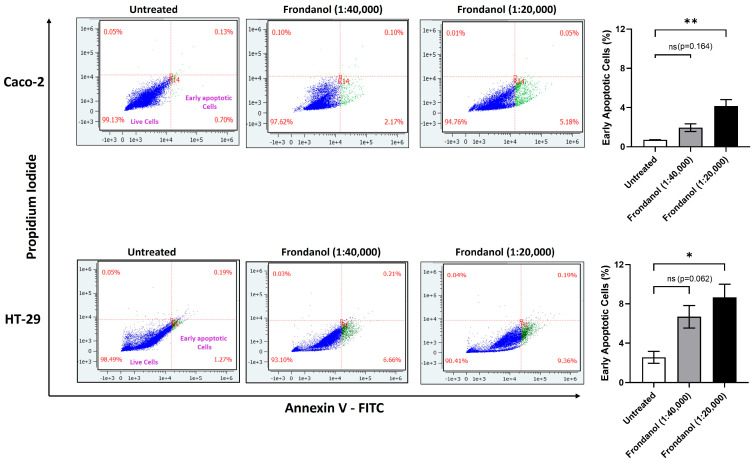
The percentage of early apoptotic cells was assessed by flow cytometry in Caco-2 (**upper panels**) and HT-29 cells (**lower panels**) using Annexin V/PI staining assay. Lower left, lower right, upper right, and upper left panels represent live, early apoptotic, late apoptotic, and necrotic cells, respectively. Each bar graph represents the mean ± SEM of three replicate experiments. * *p* < 0.05 and ** *p* < 0.01 compared to untreated; ns = non-significant.

Using flow cytometry and an annexin V-propidium iodide (PI) assay, we explored early apoptosis dynamics in Caco-2 and HT-29 cell lines after 6 h of treatment with two dilutions of Frondanol. Our findings revealed that, at a dilution of 1:40,000, there was an increase in early apoptotic cells in both Caco-2 (2.17%) and HT-29 (6.66%) compared to the untreated groups; however, this was not statistically significant. At a higher concentration (1:20,000 dilution), Frondanol significantly increased early apoptotic cells in both Caco-2 (5.18%) and HT-29 (9.36%) compared to the untreated groups (Figure 6).

### 2.7. Frondanol Inhibited Cell Proliferation Through Induction of Cell Cycle Arrest

**Figure 7:**
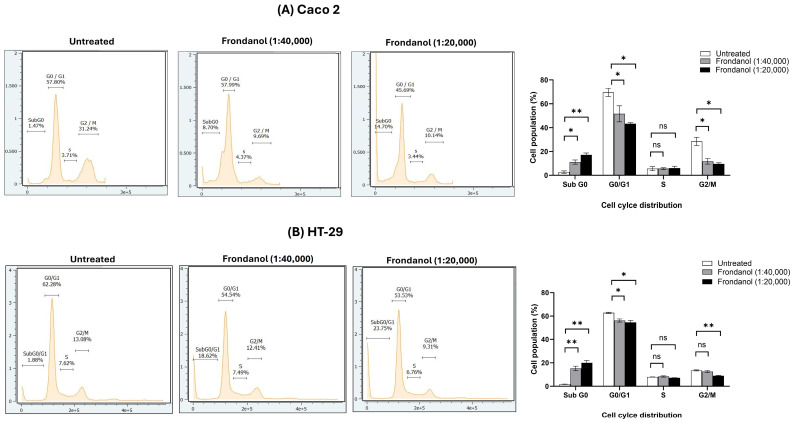
Effects of Frondanol on cell cycle distribution in (**A**) Caco-2 and (**B**) HT-29 Cells. Each bar graph represents the mean ± SEM of three replicate experiments. * *p* < 0.05, ** *p* < 0.01 compared to untreated, ns = non-significant.

Cell cycle analysis of HT-29 and Caco-2 cells treated with two concentrations of Frondanol revealed its potent antiproliferative effects, primarily by inducing apoptosis (Figure 7). In Caco-2 cells, Frondanol treatment caused a dose-dependent increase in the Sub G0 population (1.47% in untreated cells to 8.70% at 1:40,000 and 14.70% at 1:20,000), indicating apoptosis, while concurrently reducing the G0/G1 (57.80% to 45.69%) and G2/M (31.44% to 10.14%) populations. This suggests that Frondanol disrupts the cell cycle and inhibits progression through critical checkpoints. In HT-29 cells, Frondanol significantly increased the Sub G0 population (1.88% in untreated cells to 18.62% at 1:40,000 and 23.73% at 1:20,000), confirming its pro-apoptotic effect. Additionally, it decreased the G0/G1 phase (62.28% to 53.33%) and reduced the G2/M (13.08% to 9.31%) population, indicating impaired mitotic progression and inhibition of further cell division.

### 2.8. Frondanol Induced Apoptosis via Caspase Activation

**Figure 8:**
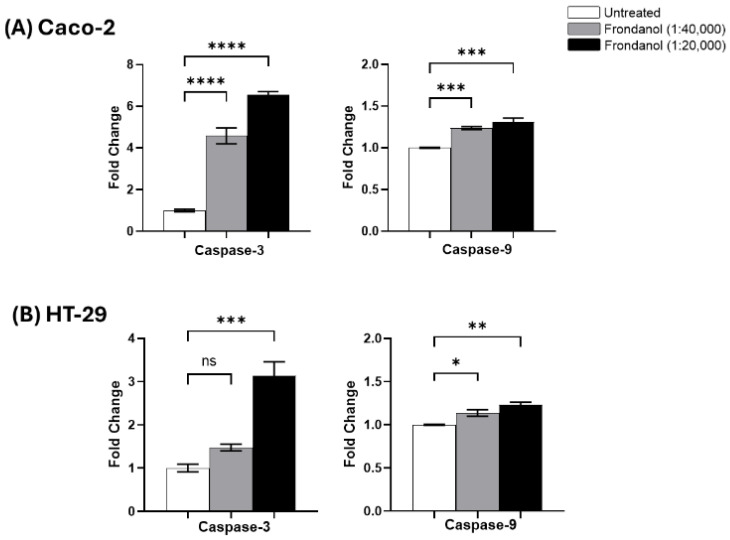
Activity of caspases 3 and 9 after treatment with Frondanol in (**A**) Caco-2 and (**B**) HT-29 cells. Each bar graph represents the mean ± SEM of three replicate experiments. * *p* < 0.05, ** *p* < 0.01, *** *p* < 0.001, **** *p* < 0.0001 compared to untreated, ns = non-significant.

To investigate whether Frondanol induced apoptosis through caspase activation, we evaluated the activities of caspase-3 and caspase-9 in Caco-2 and HT-29 cells following treatment with Frondanol. In Caco-2 cells, Frondanol treatment resulted in a 5-fold increase in caspase-3 activity at 1:20,000 and a 3-fold increase at 1:40,000 compared to untreated controls. Similarly, caspase-9 activity increased by 0.4-fold and 0.3-fold at the same concentrations, respectively (Figure 8A). In HT-29 cells, caspase-3 activity was elevated by 2-fold at 1:20,000 and 0.5-fold at 1:40,000, while caspase-9 activity increased by 0.4-fold and 0.2-fold, respectively, relative to controls (Figure 8B). These findings collectively indicate that Frondanol induces apoptosis in CRC cells by activating intrinsic apoptotic pathways.

### 2.9. Frondanol Modulated Anti-Apoptotic, Pro-Apoptotic, and Caspase Protein Expression

**Figure 9:**
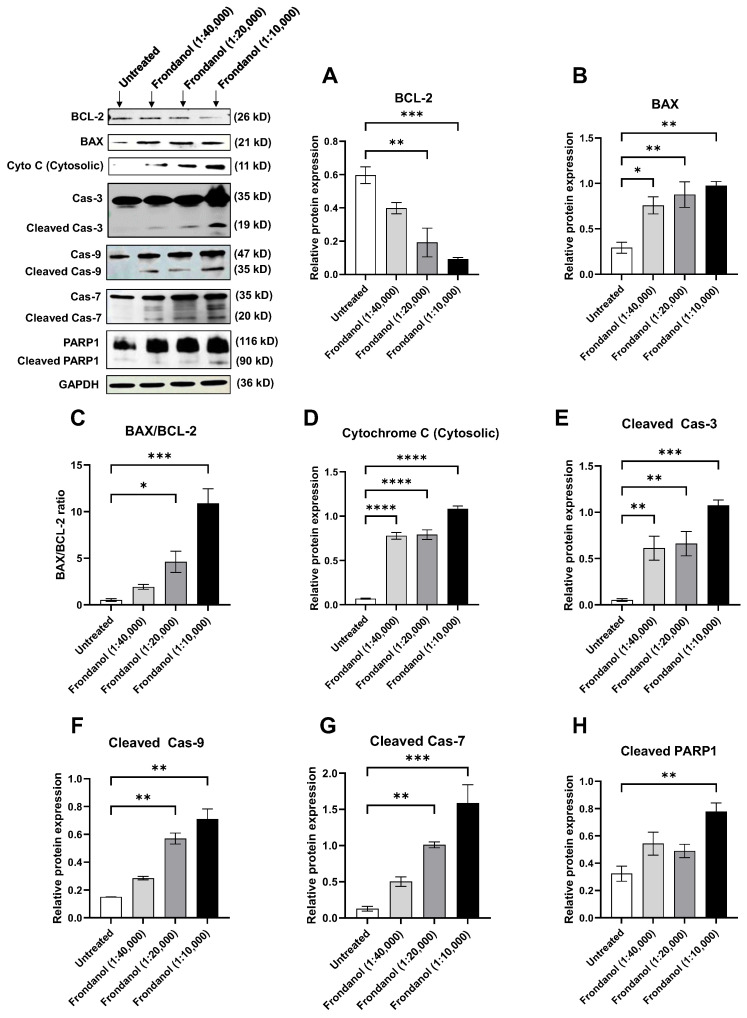
Effect of Frondanol on the relative expression of (**A**) Bcl-2, (**B**) BAX, (**C**) BAX/BCL-2 ratio, (**D**) Cytochrome C (cytosol), (**E**) Cleaved caspase-3, (**F**) Cleaved caspase-9, (**G**) Cleaved caspase-7, and (**H**) Cleaved PARP1 proteins in Caco-2 colorectal cancer cells after 48 h of treatment. Representative Western blot images of several apoptosis-related proteins are in the upper left corner. Each bar graph represents the mean ± SEM of three replicate experiments. * *p* < 0.05, ** *p* < 0.01, *** *p* < 0.001, **** *p* < 0.0001 compared to untreated.

**Figure 10:**
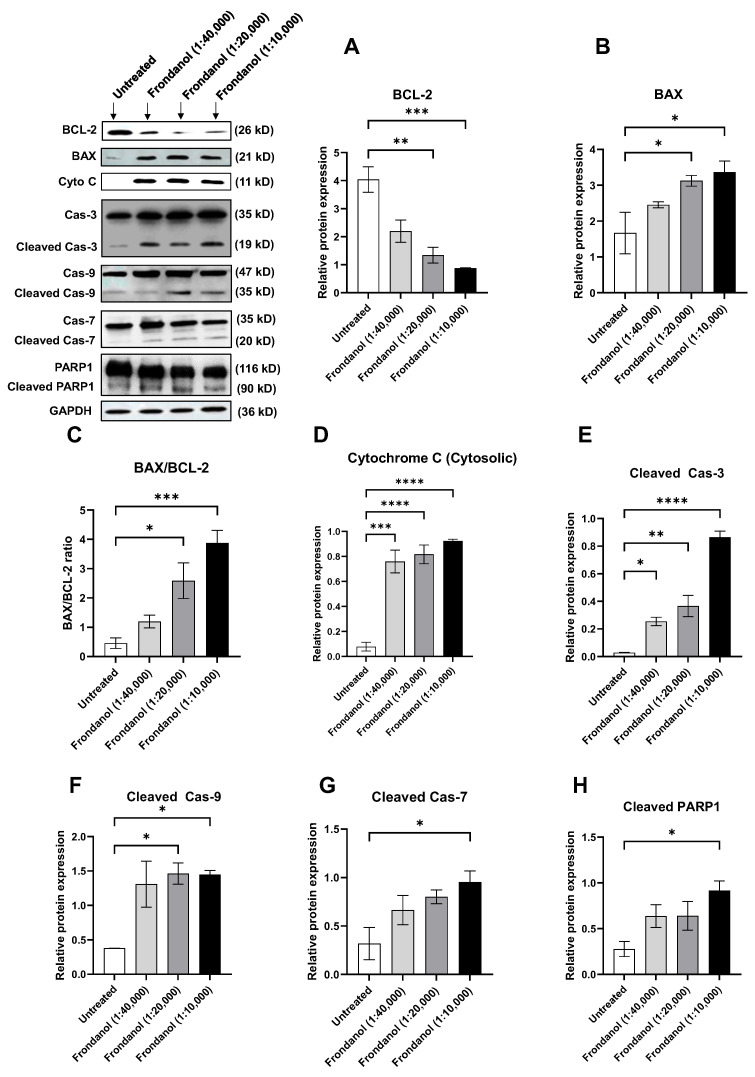
Effect of Frondanol on the relative expression of (**A**) Bcl-2, (**B**) BAX, (**C**) BAX/BCL-2 ratio, (**D**) Cytochrome C (cytosol), (**E**) Cleaved caspase-3, (**F**) Cleaved caspase-9, (**G**) Cleaved caspase-7, and (**H**) Cleaved PARP1 proteins in HT-29 colorectal cancer cells after 48 h of treatment. Representative Western blot images of several apoptosis-related proteins are in the upper left corner. Each bar graph represents the mean ± SEM of three replicate experiments. * *p* < 0.05, ** *p* < 0.01, *** *p* < 0.001, and **** *p* < 0.0001 compared to untreated.

We conducted Western blot analyses targeting key apoptotic proteins to delve into the molecular mechanisms underlying Frondanol-induced cell death. Our findings revealed a significant reduction in the levels of anti-apoptotic protein B-cell lymphoma (Bcl-2) following 48 h of Frondanol treatment in both Caco-2 and HT-29 cells (Figure 9A and Figure 10A, respectively). Conversely, Frondanol treatment significantly increased pro-apoptotic protein Bcl-2-associated X-protein (BAX) levels (Figure 9B and Figure 10B), substantially elevating the BAX/BCL-2 ratio (Figure 9C and Figure 10C). Furthermore, Frondanol treatment increased the release of cytochrome c in the cytosol of both cell lines (Figure 9D and Figure 10D), indicating disrupted mitochondrial membrane permeability. After cytochrome c release in the cytosol, activation of caspase-9 was observed, as evidenced by increased levels of active caspase-9 resulting from procaspase-9 cleavage (Figure 9F and Figure 10F). Frondanol induced marked caspase-3 activation (Figure 9E and Figure 10E) and caspase-3 substrate poly (ADP-ribose) polymerase 1 (PARP1) cleavage (Figure 9H and Figure 10H) in both Caco-2 and HT-29 cells after 48 h of treatment. Activation of caspase-7 was also seen after Frondanol treatment (Figure 9G and Figure 10G). Activation of caspase-3 led to the cleavage of its substrate PARP1, generating a cleaved fragment (Figure 9H and Figure 10H).

### 2.10. Frondanol Inhibited 5-LOX Enzyme Activity

**Figure 11:**
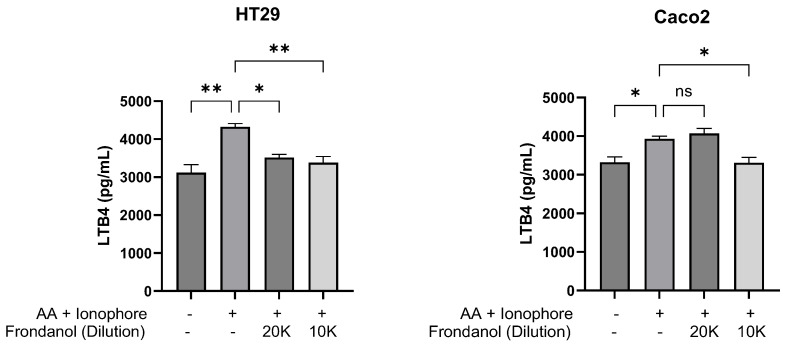
Effect of Frondanol on 5-lipoxygenase enzyme activity in HT-29 and Caco-2 colorectal cancer cells. Data are presented as mean ± SEM of three replicate experiments. * *p* < 0.05, ** *p* < 0.01 compared to untreated, and ns = non-significant. AA: arachidonic acid.

Following stimulation with arachidonic acid and calcium ionophore, Frondanol exhibited an inhibitory effect on 5-lipoxygenase (5-LOX) enzyme activity in Caco-2 and HT-29 cells, as measured by leukotriene B4 (LTB4) production. In Caco-2 cells, stimulation with arachidonic acid and ionophore increased LTB_4_ production by approximately 3.5-fold relative to the unstimulated control. Pretreatment with Frondanol at a 1:10,000 dilution reduced LTB_4_ levels by 16%, corresponding to a 1.8-fold inhibition of 5-LOX activity compared to the stimulated control. Similarly, in HT-29 cells, stimulation induced a 3.2-fold increase in LTB_4_ levels over baseline, whereas Frondanol pretreatment at 1:20,000 and 1:10,000 dilutions reduced LTB_4_ production by 20% and 22%, respectively (Figure 11).

## 3. Discussion

Frondanol is a US-patented nutraceutical oil extracted from the intestines of the edible Atlantic Sea cucumber, Cucumaria frondosa. Frondanol possesses potent anti-inflammatory properties, offering a promising avenue for combating various inflammatory diseases such as inflammatory bowel disease [10,11]. It is important to note that Frondanol is different from Frondanol A5, which is also derived from the Cucumaria frondosa but is the polar extract of its skin and is known for its anticancer properties against pancreatic cancer cells [16,17]. Frondanol suppressed inflammation in a rat model of adjuvant-induced arthritis, a mouse ear inflammatory edema model [12], and a colitis mouse model [11].

Our results showed that Frondanol inhibited cell proliferation and induced apoptosis in Caco-2 and HT-29 cells through multiple mechanisms, as confirmed by various in vitro assays. Morphological analysis revealed significant changes, including reduced cell confluence and altered cell shape, while viability assays identified IC_50_ values of approximately 1:20,000 (24 h) and 1:40,000 (48 h). Frondanol’s antiproliferative effects were further supported by its ability to inhibit colony formation, highlighting its potential to target proliferating cancer cells and prevent tumor progression. The AO/EtBr staining and Hoechst 33258 assays confirmed these pro-apoptotic effects, showing chromatin condensation and nuclear fragmentation. Mechanistic investigations revealed that Frondanol induces apoptosis through the intrinsic mitochondrial pathway. Our results indicate that Frondanol treatment increased cytochrome c (particularly in the cytosol) and induced activation of caspase-9. Furthermore, Frondanol treatment also induced enzyme activity of caspase-9. This indicates that Frondanol may induce apoptosis in CRC cells by activating a mitochondrial-mediated intrinsic pathway. The final stage of apoptosis involves activating effector caspases, including caspase-3 and caspase-7. Caspases are initially present as inactive procaspases, which are converted into active enzymes by proteolytic cleavage. These activated caspases then cleave their substrates at specific aspartic acid sites. Caspase-3, in particular, plays a vital role in this process, targeting substrates such as PARP, retinoblastoma protein, actin, and laminin [18,19]. Our results demonstrated that Frondanol treatment activated caspase-3 and caspase-7 and induced the cleavage of PARP in CRC cells. These findings indicate that Frondanol triggers the apoptosis pathway by promoting protein level activation, thus highlighting its potential as a therapeutic agent in CRC treatment. Frondanol also modulates the expression of Bcl-2 family proteins, tipping the balance towards apoptosis. Frondanol treatment significantly reduced the expression of the anti-apoptotic protein Bcl-2 while increasing the expression of the pro-apoptotic protein BAX, leading to a marked elevation in the BAX/BCL-2 ratio. This shift enhances cytochrome C release and downstream caspase activation, reinforcing the intrinsic pathway’s role in Frondanol’s anticancer activity. Moreover, Both Caco-2 and HT-29 colorectal cancer cell lines harbor mutations in the p53 gene. In Caco-2 cells, a nonsense mutation introduces a premature stop codon at position 204, resulting in the absence of detectable p53 protein expression [20,21]. In HT-29 cells, an R273H missense mutation leads to the substitution of histidine for arginine at codon 273. Although this mutation causes elevated accumulation of p53 protein, the mutant form is transcriptionally inactive and promotes increased proliferation and resistance to apoptosis [22]. Therefore, in both cell lines, mitochondrial apoptosis occurs independently of functional p53.

The inhibition of 5-LOX by Frondanol adds another dimension to its mechanism of action. Studies have linked higher fish consumption to reduced cancer risk and slower tumor growth, primarily attributed to fatty acids like EPA and DHA and the inhibition of LOX metabolites [23,24]. Consistent with literature linking omega-3–rich diets to reduced cancer risk, the GC-FID analysis of Frondanol revealed high concentrations of anti-inflammatory and antitumorigenic fatty acids—most notably EPA (17.9%). This long-chain polyunsaturated fatty acid is known to inhibit lipoxygenase-derived metabolites, suppressing tumor-promoting inflammation and slowing neoplastic progression [25,26,27]. Similarly, our GC-FID analysis of Frondanol revealed that it contains branch chained fatty acids such as 12-MTA (19.6%), which inhibit 5-LOX and 12-LOX activity. This inhibition reduces pro-inflammatory and pro-tumorigenic metabolites like LTB_4_ [12]. In vitro studies show that it suppresses proliferation of prostate cancer (PC3) cells via induction of apoptosis—marked by elevated caspase–3 activity and reduced 5-HETE levels [28]. Furthermore, in vivo evaluation using a rabbit tumor model revealed dose-dependent tumor growth inhibition, decreased 5-HETE, and increased 15-HETE levels following targeted arterial delivery of 12-MTA [29]. In this study we also have confirmed Frondanol’s 5-LOX inhibitory effects on colon cancer cells. The results showed that the Frondanol inhibited 5 LOX activity in HT-29 and Caco-2 cells as evidenced by decreased LTB_4_ production. Further supporting these findings, previous studies demonstrated that Frondanol reduced LTB_4_ concentrations in colonic tissue from a dextran sulfate sodium-induced mouse model of inflammatory bowel disease [11]. In nutshell, presence of EPA and 12-MTA in Frondanol may contribute significantly to the observed anticancer effects in colon cancer cells. Both compounds are known to inhibit lipoxygenase pathways and reduce pro-inflammatory eicosanoids, thereby suppressing tumor-promoting signaling and enhancing apoptosis.

The results of this study hold significant clinical relevance, particularly for developing novel CRC therapies. Frondanol’s ability to induce apoptosis through intrinsic pathways and its inhibition of 5-LOX activity underscores its potential as a dual-action therapeutic agent. This dual mechanism offers a targeted approach that disrupts tumor cell survival and addresses inflammation-related pathways implicated in CRC progression. Compared to conventional chemotherapeutic agents, Frondanol may provide a more effective and potentially less toxic alternative. Similar observations have been made with other marine-derived compounds, further supporting their potential to enhance cancer treatment outcomes [16,17,30,31].

Despite the promising findings, several limitations must be addressed. The current study focuses on in vitro models, and the results need validation in vivo to confirm Frondanol’s efficacy and safety in a physiological context. Long-term studies assessing its potential toxicity and pharmacokinetics are crucial for clinical translation. Additionally, exploring the combinatory effects of Frondanol with existing chemotherapeutic agents could enhance its therapeutic efficacy while minimizing resistance. Previous research on marine-derived or plant-derived anticancer compounds has highlighted the benefits of such combinatory approaches, warranting further investigation into Frondanol’s potential in combination therapies [32,33,34].

In conclusion, our study demonstrates that Frondanol, a nutraceutical derived from the edible Atlantic sea cucumber *Cucumaria frondosa*, exhibits significant anticancer effects on CRC cells. Frondanol effectively reduces cell viability, inhibits colony formation, and induces morphological changes indicative of apoptosis, highlighting its potential as a treatment for CRC. Mechanistic investigations show that Frondanol triggers apoptosis through the mitochondrial-mediated intrinsic pathway, evidenced by a significant increase in the BAX/BCL-2 ratio, which promotes cytochrome c release, caspase activation, and PARP cleavage. This effect may also be partially linked to the inhibition of 5-LOX. The dual modulation of pro-apoptotic and anti-apoptotic proteins emphasizes Frondanol’s potential as a therapeutic agent in CRC. Furthermore, EPA and 12-MTA, identified through fatty acid profiling of Frondanol, are likely key contributors to its observed preventative effects against colorectal cancer, supporting its potential as a bioactive anticancer agent. Fatty acid profiling identified 12-MTA as the major bioactive component that may contribute to Frondanol’s observed anticancer effects. As part of our future work, we aim to (i) evaluate the anticancer activity of purified 12-MTA as a proof of concept and (ii) perform in vivo validation studies in colorectal cancer animal models to substantiate and translate these in vitro findings.

## 4. Materials and Methods

### 4.1. Frondanol and Its Preparation

Frondanol is a trademarked nutraceutical lipid extract (developed and marketed by Coastside Bio Resources, Deer Isle, ME, USA) derived from the gut material of edible Atlantic Sea cucumber (*Cucumaria frondosa*). The extraction process involves using hexane as an organic solvent to isolate lipids from the intestinal tissues of the sea cucumber. Frondanol, in the form of oil, is encapsulated within soft-gel capsules. For the current investigation, the oil contained in the capsules was initially dissolved in dimethyl sulfoxide (DMSO) to prepare a working solution. This DMSO-dissolved oil was then further diluted in Dulbecco’s Modified Eagle’s Medium (DMEM/F-12) (Sigma, Louis, MO, USA)to produce a series of dilutions: 1:10,000, 1:20,000, 1:40,000, and 1:80,000, corresponding to final concentrations of 0.01%, 0.005%, 0.0025%, and 0.00125% (*v*/*v*), respectively. These dilution ranges were selected based on our prior work, which demonstrated effective bioactivity of Frondanol within this concentration window [12,13]. To achieve maximum solubility, each dilution step was carried out using warm (37 °C) conditions, followed by vigorous mixing and brief sonication to ensure the oil remained suspended.

### 4.2. Gas Chromatography-Flame Ionization Total Fatty Acid Analysis of Frondanol

The fatty acid profile of Frondanol was determined by Eurofins Scientifics (Castle Rock, CO, USA) using gas chromatography with flame ionization detection (GC-FID). Fatty acids were first liberated from triglycerides and complex lipids through saponification and subsequently converted into fatty acid methyl esters (FAMEs) via transesterification. An internal standard (e.g., C13:0 or C19:0) was added before extraction to ensure accurate quantification. FAMEs were separated using a wax-type capillary column, and peaks were identified and quantified based on retention times. Results were reported as weight percent of individual fatty acids relative to the total sample. This method allowed the quantification of saturated, monounsaturated, and polyunsaturated fatty acids.

### 4.3. Cell Lines and Cultures

Two CRC cell lines, Caco-2 and HT-29, purchased from AddexBio (San Diego, CA USA) were cultured with DMEM/F-12 supplemented with 100 U/mL streptomycin, 100 U/mL penicillin (Gibco, Life Technologies, Massachusetts, MA, USA), and 10% fetal bovine serum (Biowest, Bradenton, FL, USA) and incubated in 5% carbon dioxide at 37 °C. The culture flasks were sub cultured every 48 to 72 h, and cells were transferred into the new flasks when almost fully grown at 70–80% confluence.

### 4.4. Cell Viability Assay and Morphological Analysis

Cell viability based on ATP measurement was evaluated using CellTiter-Glo Luminescent Cell Viability Assay (Promega, Madison, WI, USA). Briefly, cells (5000 cells per well) were seeded in a 96-well plate and incubated overnight in a CO_2_ incubator at 37 °C. The next day, cells were shifted to serum-free media for 24 h serum starvation. After that, cells were treated with different dilutions (1:80,000, 1:40,000, 1:20,000, and 1:10,000 (*v*/*v*)) of Frondanol for 24 and 48 h. Untreated cells served as normal control, while cells treated with DMSO (1%) only were vehicle-controlled. On the day of the assay, cell culture media was removed from each well and replaced with 100 µL of fresh media. An equal volume of the Cell Titer-Glo^®^ reagent was added to each well and mixed on an orbital shaker for two minutes to enhance cell lysis. The plate was incubated for 10 min at room temperature, and the luminescent signal was recorded using a microplate reader (Hidex Sense 425-311, Turku, Finland). The morphological changes in treated and untreated colorectal cancer cells were examined via an inverted phase-contrast microscope (Olympus, CKX53, Tokyo, Japan) at 40× magnification.

### 4.5. Clonogenic Assay

Clonogenic cell death was measured using a colony-forming assay according to the method of Franken and colleagues [35]. The clonogenic assay was employed to evaluate cell survival. Approximately 500 cells per well were seeded on a six-well plate and allowed to grow as colonies (with a minimum of 50 cells per colony) for 14 days. Subsequently, the growth medium was replaced daily with or without Frondanol for seven days. Following the seven-day treatment, the colonies were washed with phosphate-buffered saline (PBS) and fixed in a fixative buffer (methanol: acetic acid = 7:1) for 15 min at room temperature on a shaker. After fixation, colonies were washed with PBS and stained with crystal violet (0.5%, *w*/*v* prepared in 25% methanol) for 3 h at room temperature on the shaker. Finally, the colonies were photographed using an inverted microscope (Olympus CKX41, Tokyo, Japan) and counted using ImageJ software (1.54g).

### 4.6. Qualitative Staining for Apoptosis Assessment

Qualitative staining techniques, such as acridine orange (Med Chem Express, NJ, USA)/ethidium bromide (AO/EtBr) and Hoechst 33342 (Invitrogen, Thermo Fisher Scientific, Waltham, MA, USA) staining, are commonly employed to assess apoptosis. These methods involve staining cells with dyes that selectively target apoptotic features, providing visual evidence of cellular changes indicative of apoptosis.

1. AO/EtBr double staining

AO/EtBr double staining was utilized to explore the apoptosis triggered by Frondanol in colorectal cancer cells. This technique effectively identifies normal, early, late apoptotic, and dead cells. Ethidium bromide selectively labels cells with damaged cell membranes, while acridine orange permeates living and dead cells, imparting a green hue to the nucleus. Thus, viable cells display consistent green staining. In contrast, apoptotic cells manifest orange or red staining with acridine orange, contingent upon the extent of membrane impairment and concurrent staining with ethidium bromide [36]. Briefly, 5000 cells were plated onto 12 well chamber slides (ibidi GmbH, Gräfelfing, Germany), and upon reaching confluence, they were subjected to treatment with Frondanol at 1:20,000 dilution for 24 h at 37 °C, following established protocol [36]. After 24 h of treatment, adherent cells were gently washed with PBS, and then 50 μL of a dye mixture containing EtBr (1 mg/mL) and AO (1 mg/mL) was added to each chamber well. The chamber slides were promptly examined using a fluorescence microscope, and images were captured using (FITC filter for AO and TRITC filter for EtBr) a Nikon D 700 camera connected to the microscope (Olympus CKX41, Tokyo, Japan).

2. Hoechst 33342 staining

The Hoechst 33342 dye assay was used to study the nuclear condensation in Frondanol-treated colorectal cancer cells, as described by Harada et al. [37]. Cells were seeded in an eight-well chamber slide and treated with Frondanol (1:20,000) for 48 h. The cells were then rinsed in PBS, fixed for 15 min in acetic acid and methanol (1:3), and stained for 20 min in the dark at 37 °C with Hoechst 33342 (5 µg/mL). The excess stain was removed by washing with PBS and viewed under the fluorescence microscope. Images were captured using a DAPI filter and a Nikon D 700 camera connected to the microscope (Olympus CKX41, Tokyo, Japan).

### 4.7. Early Apoptosis Assay (Annexin V/Propidium Iodide Assay)

Examining membrane asymmetry is a vital indicator of cell viability, reflecting the uneven distribution of phospholipids across the membrane layers. In early apoptosis, the relocation of phosphatidylserine from the inner to the outer plasma membrane disrupts membrane phospholipid symmetry. With its affinity for negatively charged phosphatidylserine, annexin V proves invaluable in detecting early apoptosis through techniques such as flow cytometry. Cells in early apoptosis that bind annexin V do not exhibit intracellular staining with propidium iodide. However, as apoptosis progresses, the integrity of the plasma membrane deteriorates, facilitating the entry of propidium iodide into cells, where it strongly binds to the DNA double helix [38,39]. The early apoptosis rate was measured using a flow cytometer with an eBioscience™ Annexin V Apoptosis Detection Kit (Invitrogen, Waltham, MA, USA). Cells were seeded in 6-well plates at a density of 1 × 10^5^ cells per well in 2 mL of culture media and treated with or without Frondanol for 6 h. Following treatment, cells were trypsinized and washed with PBS, followed by an additional wash with a binding buffer (provided in the kit) to ensure proper cellular preparation. The washed cells were then resuspended in a binding buffer at a concentration ranging from 1 to 5 million cells per mL. Subsequently, 2 µL of fluorochrome-conjugated Annexin V was added to 100 µL of the resuspended cell suspension, and the mixture was incubated for 25 min at room temperature in the dark. Following Annexin V staining, cells were washed again with binding buffer and resuspended in 150 µL of the same buffer. Then, 2 µL of propidium iodide (PI) was added to the cell suspension, and the mixture was incubated for 15 min at room temperature in the dark. The stained cell samples were analyzed using a flow cytometer (Amnis^®^ CellStream^®^ Flow Cytometer, Luminex Corporation, Austin, TX, USA). Flow cytometry data were collected and analyzed using CellStream^®^ Analysis 1.3.384 software.

### 4.8. Caspase Activity Assays

Caspase activity assays conducted in multi-well plate formats are valuable tools for investigating how experimental interventions impact the apoptotic response. We utilized the Caspase 3 and Caspase 9 Multiplex Activity Assay Kit (Abcam, Cambridge, UK), which employs fluorescence to detect the activity of these caspases. Briefly, cells were plated in 96-well plates at a density of 2 × 10^4^ cells/well in 100 μL of culture medium and treated with different concentrations of Frondanol for 48 h for caspase 3 and 24 h for caspase 9. Subsequently, 100 μL of substrate-specific to each caspase was added to the cells. Following a 50 min incubation period, the fluorescence intensity was measured using a fluorescence microplate reader (Hidex Sense 425-311, Turku, Finland) at specific wavelengths: 535/620 nm for Caspase 3 and 370/450 nm for caspase-9. The findings were presented in fold change as an elevation in caspase 3 and 9 activities compared to the control.

### 4.9. Cell Cycle Analysis

Colorectal cancer cells HT-29 and Caco-2 cells were seeded at a density of 1 × 10^5^ cells per well in 2 mL of culture media in 6 well plates. Following overnight incubation and 24 h serum starvation, the cells were treated with varying concentrations of Frondanol for 48 h. Post treatment, the cells were trypsinized, centrifuged at 300× *g* for 5 min, and washed once with cold PBS. Subsequently, the cells were fixed in ice-cold 70% ethanol (added dropwise) and incubated at 4 °C for an hour. After fixation, the cells underwent centrifugation at 450× *g* for 5 min and were washed once with cold PBS. Following this, 200 μL of Guava^®^ Cell Cycle (Cytek Biosciences, CA, USA) reagent (diluted with distilled water 1:4) was added to each tube, and the tubes were incubated for 30 min at 37 °C in the dark to enable staining. Analysis was conducted using a flow cytometer (Amnis^®^ CellStream^®^ Flow Cytometer). Flow cytometry data were collected and analyzed using CellStream^®^ Analysis 1.3.384 software.

### 4.10. Western Blotting

CRC cells (HT-29 and Caco-2) were seeded (3 × 10^5^ cells/well) in a 6-well plate and incubated for 24 h; then, they were treated with different dilutions of Frondanol and incubated for 48 h. After that, the cells were lysed using RIPA lysis buffer containing a phosphatase and protease inhibitor cocktail and centrifuged at 14,000 rpm, at 4 °C, for 25 min to separate cell proteins. To measure cytochrome C protein expression in the cytosol, cell pellets from untreated and Frondanol-treated cells were suspended in cytosol extraction buffer (220 mM mannitol, 68 mM sucrose, 50 mM HEPES-KOH (pH 7.4), 50 mM KCl, 5 mM EGTA, 2 mM MgCl2, 1 mM DTT, and protease inhibitors). After 30 min of incubation on ice, cells were homogenized by passing through a 1 mL syringe using a 27-gauge needle (at least ten times). Cell homogenates were spun at 14,000× *g* for 15 min. The supernatants (cytosolic fraction) were used to measure cytochrome C protein expressions [40]. The extracted proteins were quantified through a Pierce™ BCA Protein assay kit (Thermo Fisher, Waltham, MA, USA), and 20–50 µg was electrophoresed on an acrylamide gel (4–15% Mini-PROTEAN^®^ TGX™ Precast; Bio-Rad, Hercules, CA, USA). The separated proteins were transferred to a nitrocellulose membrane. Then, the membrane and primary antibodies were left to react overnight at 4 °C on the shaker. After that, the membrane was left to react with the secondary antibody for one hour at room temperature; then, it was washed sufficiently, and the protein expression level was obtained using a chemiluminescent substrate (Thermo Fisher, Waltham, MA, USA). Western blot results were quantified using ImageJ (National Institutes of Health, Bethesda, MD, USA).

### 4.11. 5-LOX Enzyme Activity

CRC cells, HT-29 and Caco-2, were seeded at a density of 25,000 cells per well in a 96-well plate and incubated overnight. The following day, the cells were treated with Frondanol at 1:10,000 and 1:20,000 dilutions for 30 min. After the 30 min treatment, 10 µM of arachidonic acid was added to each well, excluding the control wells, and the cells were incubated for 5 min. Subsequently, 5 µM of calcium ionophore (A23187) was added to the wells (except control wells) and incubated for 5 min. At the 40 min mark, 100 µL of the cell supernatant was collected from each well, and LTB4 levels were measured using an ELISA (ELK Biotechnology, Denver, CO, USA) kit according to the manufacturer’s instructions.

### 4.12. Statistical Analysis

Statistical analyses were conducted using GraphPad Prism (version 10.2.1.) software (San Diego, CA, USA). Differences among treatment groups and untreated groups were assessed using a one-way analysis of variance (ANOVA) followed by Dunnett’s post hoc multiple comparison test. A significance level of *p* < 0.05 was considered statistically significant.

## Figures and Tables

**Table 1:** Fatty acid profile of Frondanol.

Fatty Acid	% Weight
12-Methyltetradecanoic acid (12-MTA)	19.6
C08:0 Octanoic (Caprylic)	<0.1
C10:0 Decanoic (Capric)	<0.1
C11:0 Undecanoic (Hendecanoic)	<0.1
C12:0 Dodecanoic (Lauric)	<0.1
C14:0 Tetradecanoic (Myristic)	2.54
C14:1 Tetradecenoic (Myristoleic)	2.71
C15:0 Pentadecanoic	1.07
C15:1 Pentadecenoic	<0.1
C16:0 Hexadecanoic (Palmitic)	1.97
C16:1 Hexadecenoic (Palmitoleic)	12.7
C16:2 Hexadecadienoic	<0.1
C16:3 Hexadecatrienoic	<0.1
C16:4 Hexadecatetraenoic	<0.1
C17:0 Heptadecanoic (Margaric)	0.56
C17:1 Heptadecenoic Margaroleic	<0.1
C18:0 Octadecanoic (Stearic)	2.84
C18:1 Octadecenoic (Oleic)	4.49
C18:2 Octadecadienoic (Linoleic)	2.54
C18:3 Octadecatrienoic (Linolenic)	0.28
C18:4 Octadecatetraenoic	<0.1
C19:0 Nonadecanoic	<0.1
C20:0 Eicosanoic (Arachidic)	0.33
C20:1 Eicosenoic (Gadoleic)	0.39
C20:2 Eicosadienoic	<0.1
C20:3 Eicosatrienoic	0.55
C20:4 Eicosatetraenoic (Arachidonic)	0.60
C20:5 Eicosapentaenoic (EPA)	17.9
C21:0 Heneicosanoic	<0.1
C21:5 Heneicosapentaenoic	<0.1
C22:0 Docosanoic (Behenic)	0.21
C22:1 Docosenoic (Erucic)	0.30
C22:2 Docosadienoic	0.80
C22:3 Docosatrienoic	<0.1
C22:4 Docosatetraenoic	<0.1
C22:5 Docosapentaenoic	0.46
C22:6 Docosahexaenoic (DHA)	0.60
C24:0 Tetracosanoic (Lignoceric)	<0.1
C24:1 Tetracosenoic (Nervonic)	0.99
Unidentified Fatty acids	25.6

**Table 2:** Fatty acid composition in Frondanol.

Fatty acid Composition	% Weight
Saturated	9.52
Monosaturated	21.68
Polyunsaturated	23.73

## Data Availability

The original contributions presented in the study are included in the article, further inquiries can be directed to the corresponding author.
